# Construction of a Novel Immune-Related mRNA Signature to Predict the Prognosis and Immune Characteristics of Human Colorectal Cancer

**DOI:** 10.3389/fgene.2022.851373

**Published:** 2022-03-23

**Authors:** Jianxin Li, Ting Han, Xin Wang, Yinchun Wang, Xuan Chen, Wangsheng Chen, Qingqiang Yang

**Affiliations:** Department of General Surgery (Gastrointestinal Surgery), the Affiliated Hospital of Southwest Medical University, Luzhou, China

**Keywords:** colorectal cancer, immune, mRNA, prognosis, immunotherapy

## Abstract

**Background:** Anti-cancer immunotherapeutic approaches have gained significant efficacy in multiple cancer types. However, not all patients with colorectal cancer (CRC) could benefit from immunotherapy due to tumor heterogeneity. The purpose of this study was to construct an immune-related signature for predicting the immune characteristics and prognosis of CRC.

**Methods:** RNA-sequencing data and corresponding clinical information of patients with CRC were obtained from The Cancer Genome Atlas (TCGA) and Gene Expression Omnibus (GEO), and immune-related genes (IRGs) were downloaded from the Immunology Database and Analysis Portal (ImmPort). Then, we utilized univariate, lasso regression, and multivariate cox regression to identify prognostic IRGs and develop the immune-related signature. Subsequently, a nomogram was established based on the signature and other prognostic factors, and its predictive capacity was assessed by receiver operating characteristic (ROC) and decision curve analysis (DCA). Finally, associations between the signature and the immune characteristics of CRC were assessed.

**Results:** In total, 472 samples downloaded from TCGA were divided into the training cohort (236 samples) and internal validation cohort (236 samples), and the GEO cohort was downloaded as an external validation cohort (122 samples). A total of 476 differently expressed IRGs were identified, 17 of which were significantly correlated to the prognosis of CRC patients. Finally, 10 IRGs were filtered out to construct the risk score signature, and patients were divided into low- and high-risk groups according to the median of risk scores in the training cohort. The high-risk score was significantly correlated with unfavorable survival outcomes and aggressive clinicopathological characteristics in CRC patients, and the results were further confirmed in the internal validation cohort, entire TCGA cohort, and external validation cohort. Immune infiltration analysis revealed that patients in the low-risk group infiltrated with high tumor-infiltrating immune cell (TIIC) abundances compared to the high-risk group. Moreover, we also found that the immune checkpoint biomarkers were significantly overexpressed in the low-risk group.

**Conclusion:** The prognostic signature established by IRGs showed a promising clinical value for predicting the prognosis and immune characteristics of human CRC, which contribute to individualized treatment decisions.

## Introduction

Colorectal cancer (CRC), known as the third most frequently diagnosed malignancy worldwide, is the second leading cause of cancer-related death (Keum and Giovannucci, 2019). It is reported that more than 1.8 million newly diagnosed CRC cases and 0.8 million cancer-related deaths occurred in 2018 (Bray et al., 2018). Currently, combining surgical resection with chemotherapy is the most effective way to treat CRC. However, local recurrences and remote metastases of CRC are difficult to prevent, which is responsible for the poor prognosis of patients with CRC (Inadomi, 2017). Therefore, identifying other effective treatments to combat CRC and improve the prognosis of patients with CRC is an urgent need.

Immune cells in the tumor microenvironment (TME) can induce the host immune response to suppress the growth of tumor cells by secreting cytokines, cytokine receptors, and other factors (Ge et al., 2019). However, studies have reported that the immune system is frequently dysregulated in TME and inextricably associated with the progression of tumor cells (Gessani and Belardelli, 2019). Immune escape is a significant characteristic of tumor cells that prompts the advent of immunotherapy, a novel kind of therapy for cancer treatment (Vilgelm and Richmond, 2019). Anti-cancer immunotherapeutic approaches, including immune checkpoint inhibitors (ICIs), adoptive cell therapy, monoclonal antibodies, and cancer vaccines, have gained significant efficacy in multiple cancer types (Markman and Shiao, 2015). Among them, the immune checkpoint can terminate the immune responses and induce the failure of immune cells, thereby inducing the immune evasion of tumor cells in the TME (Pardoll, 2012). Recently, immune checkpoints such as programmed cell death 1 (PD-1) and its ligand PD-L1 and cytotoxic T-lymphocyte-associated antigen 4 (CTLA-4) received the most attention. Studies have reported that blockade of these immune checkpoints by ICIs is superior to traditional treatments and is an effective way to improve the prognosis of oncologic patients (Wang et al., 2017). However, accumulating evidence has found that the majority of CRC patients do not benefit from existing immunotherapy, and the molecular mechanism remains largely unknown (Ciardiello et al., 2019). The molecular profiles of the immune components within the TME represent a promising value in serving as a predictor of prognosis and responsiveness to immunotherapy in tumor. Therefore, identifying novel biomarkers to predict the immune characteristics is critical for evaluating the prognosis and immunotherapeutic sensitivity of CRC. Recently, numerous studies have focused on developing a prognostic signature based on molecular profiles of the immune components and found that the signature could serve as an independent predictor in CRC (Zhu et al., 2020), (Li et al., 2021). However, the precision of the predictive immune-related signature needed to be improved and external validation was needed. In addition, the associations between the identified signature and immune cell phenotypes should be illustrated to identify which group of CRC patients is more likely to benefit from immunotherapy.

In the present study, we systematically analyzed the transcriptome data and the corresponding clinical information of CRC patients based on TCGA and Gene Expression Omnibus (GEO) databases. As a result, we developed a prognostic scoring system based on 10 immune-related genes (IRGs) and verified the predictive accuracy with internal and external cohorts. In addition, we further illustrated the correlations of the IRG signature with immune cell phenotypes and immune checkpoint biomarkers in CRC. These findings revealed that the IRG signature could serve as a reliable predictor of prognosis and immune characteristics in human CRC, which contribute to individualized treatment decisions.

## Materials and Methods

### Data Acquisition and Processing

The RNA-sequencing profile data in the format of Fragments Per Kilobase Million (FPKM) of 612 CRC samples (including 44 normal samples and 568 tumor samples) were obtained from The Cancer Genome Atlas (TCGA, https://tcga-data.nci.nih.gov/tcga/). Meanwhile, the clinical information of 548 CRC cases was also downloaded from TCGA. The samples with incomplete survival information were excluded, and finally, 472 samples with complete follow-up data were included in the next analysis. In addition, RNA-sequencing profile data and corresponding clinical information of 122 samples from Gene Expression Omnibus (GEO, GSE38832, https://www.ncbi.nlm.nih.gov/geo) database were downloaded as a validation cohort. IRGs were obtained using the Immunology Database and Analysis Portal (ImmPort, http://www.immport.org) database, a powerful online platform that provides a mass of immune-related molecular profiles in different tumor types (Bhattacharya et al., 2014).

### Identification of Differentially Expressed Immune-Related mRNAs

We used the “limma” package in R to identify differentially expressed genes (DEGs) between tumor and normal samples in TCGA (Ritchie et al., 2015). The threshold for the differentially expressed gene was set as adjusted *p* value < 0.05 combined with | log2(fold change) | ≥1. Then, based on the ImmPort database, we obtained the expression profile of differentially expressed IRGs in CRC using the “VennDiagram” package in R (Chen and Boutros, 2011). To better understand the biological function of these differentially expressed IRGs in human CRC, we performed Gene Ontology (GO) and Kyoto Encyclopedia of Genes and Genomes (KEGG) enrichment analyses on these genes via the “clusterProfiler” packages in R (Yu et al., 2012). Only the enrich terms with *p* < 0.05 were considered statistically significant.

### Establishment of a Risk Signature to Evaluate the Risk Score

First, all the CRC samples obtained from TCGA were randomly assigned into training and internal validation cohorts at a 1:1 ratio. Then, we used a univariate Cox regression model to identify the association between immune-related mRNAs in relation to the prognosis of CRC patients. IRGs with *p* < 0.01 in univariate Cox analysis were incorporated into the least absolute shrinkage and selection operator (LASSO) model to prevent overfitting, and the results of LASSO analysis were included into a multivariate Cox model to construct a risk signature. Ultimately, we constructed a prognostic IRG signature, and the risk score of each patient was calculated as follows: = Ʃ (βi × EXPi), where βi (coefficients) represented the weight of the respective signature and EXPi represented the expression level. Based on the median of the risk score, CRC patients in the training cohort were divided into the low- and high-risk groups. In addition, the internal validation cohort and entire cohort were also classified into low- and high-risk groups according to the same cutoff value.

### Survival Analysis of Risk Score Signature

In order to evaluate the prognostic value of the IRG signature, the survival difference between the low- and high-risk groups was illustrated by Kaplan–Meier analysis with the log-rank test. The area under the curve (AUC) in the receiver operating characteristic (ROC) curve was drawn to verify the diagnostic efficacies of the IRG signature based on the “survivalROC” package in R (Heagerty et al., 2000). In addition, the predictive value of the IRG signature was also assessed in the internal validation and entire cohorts. Furthermore, we also performed stratified survival analysis to further determine the predictive value of the IRG signature in distinct subgroups in the entire cohort.

### External Validation of the Risk Score

To determine whether the IRG signature had a similar predictive value in different populations, its prognostic capability was further validated using the GEO cohort. The same prognostic scoring system developed in the training cohort was used to calculate the risk score for each patient in the GEO cohort, and patients were also divided into low- and high-risk groups based on the median of risk score. Then, survival curve, risk curve, and ROC curve analysis were performed.

### Clinical Parameter Correlation Analysis

To evaluate the clinical relevance of the developed IRG signature, we first obtained the clinicopathological information from TCGA, including age, gender, American Joint Committee on Cancer (AJCC) tumor stage, T classification, N classification, and M classification. Then, the differences of these clinicopathological features between low- and high-risk groups were evaluated using the Chi-square test. In addition, we performed the Wilcoxon test to assess the risk score differences across different subgroups based on the clinicopathological characteristics.

### Construction of a Predictive Nomogram

We constructed a nomogram consisting of clinical parameters and risk score based on multivariate Cox regression analysis using “survival,” “rms,” and “foreign” packages in R software. The calibration curve was plotted to compare the association between the actual outcomes and the predicted probabilities. The AUC of the ROC curve was plotted to compare the predictive abilities of the nomogram with other prognostic factors. Furthermore, we used decision curve analysis (DCA) to evaluate the net benefit of the nomogram in a clinical context.

### Comparison of the IRG Signature With Other Prognostic Models

To determine whether our IRG signature is better than previously identified models in CRC, we compared the predictive ability of our IRG signature with other signatures, including a six-mRNA signature (Dai et al., 2020), a five-mRNA signature (Zhu et al., 2020), and two eight-mRNA signature (Wang et al., 2020; Wu et al., 2020). We obtained the IRGs in these signatures from the corresponding published literature, and then calculated the C-index and AUC of 1-, 3-, and 5-year ROC curves for each signature.

### Estimation of Tumor-Infiltrating Immune Cells and Immunotherapeutic Biomarkers With Risk Assessment Signature

To explore the correlation between the risk score and TIIC characteristics, we applied the currently developed deconvolution algorithm, including TIMER (Li et al., 2017), QUANTISEQ (Plattner et al., 2020), MCPcounter (Dienstmann et al., 2019), EPIC (Racle et al., 2017), and CIBERSORT (Chen et al., 2018) to calculate the abundances of distinct TIICs in each sample from the TCGA project. We developed a lollipop diagram to show the correlation between the risk score and TIIC abundance based on Spearman correlation analysis, and the difference in TIIC abundance between low- and high-risk groups was analyzed using the Wilcoxon test and shown in a box plot. In addition, we compared the expression levels of immunotherapeutic biomarkers between low- and high-risk groups based on the Wilcoxon test.

### Statistical Analysis

All statistical analyses were performed using R software (version 4.0.2). The Student’s t-test was applied to identify statistically differentially expressed genes, and the Wilcox test was used to identify statistically differentially infiltrative immune cells. Fisher’s exact test was used to evaluate the significant GO terms and KEGG enrichment pathways. Cox regression and Kaplan–Meier curves with the log-rank test were used for evaluating the survival data. The ROC curve was used to determine the predictive accuracy of the risk signature, and the DCA and calibration curves were used to explore the reliability of the nomogram. The correlation between the risk score and TIIC abundance was evaluated using the Spearman correlation coefficient. *p* value < 0.05 was considered statistically significant.

## Results

### Identification of Differentially Expressed Immune-Related mRNAs

**FIGURE 1:**
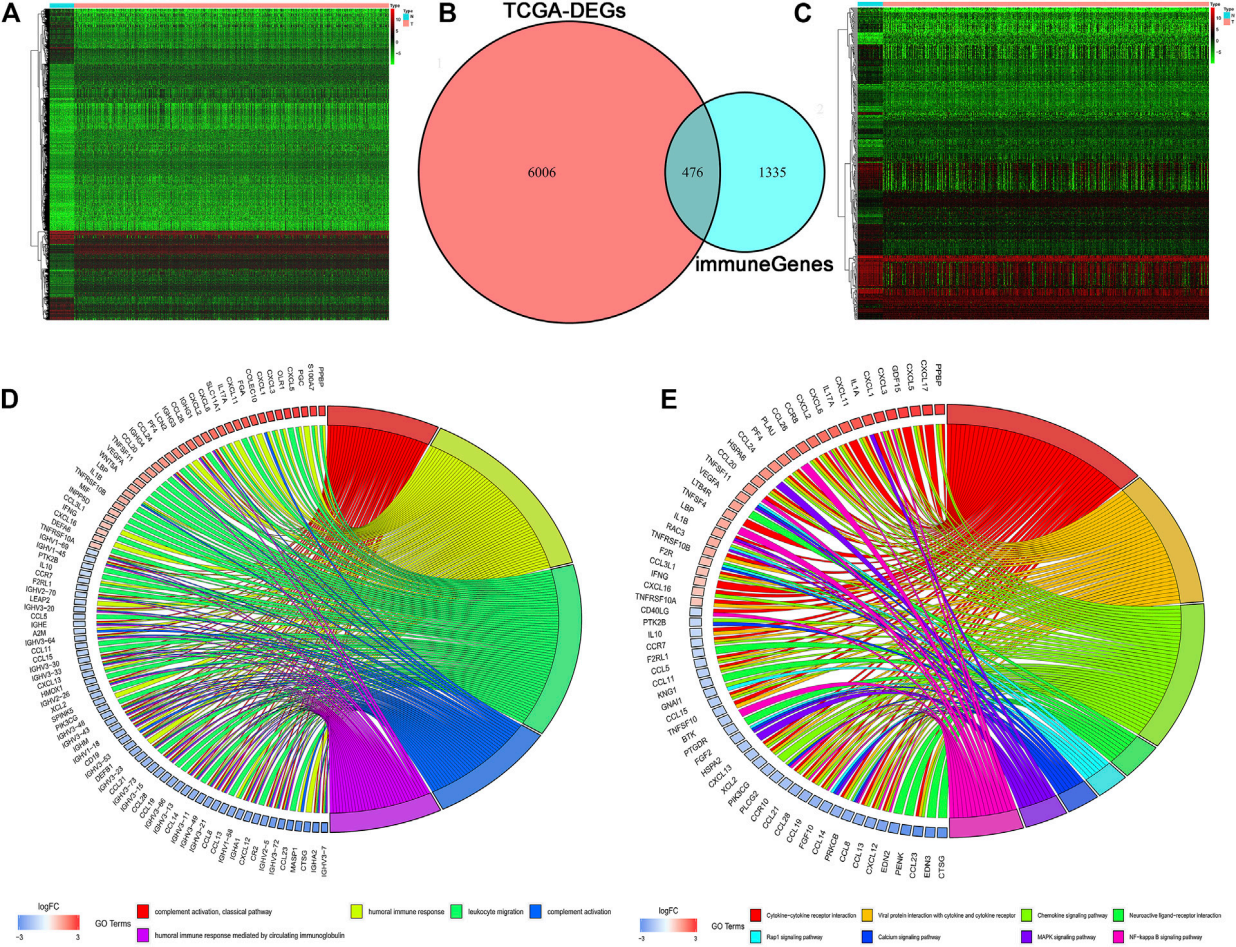
Identification of differentially expressed IRGs. **(A)** The heatmap of differentially expressed genes in TCGA. **(B)** The intersection of differentially expressed genes and IRGs. **(C)** The heatmap of differentially expressed IRGs. **(D, E)** GO and KEGG enrichment analyses of differentially expressed IRGs.

In total, 6,482 DEGs including 4,518 upregulated and 1,964 downregulated genes were identified based on the threshold of adjusted *p* value < 0.05 and | log2(fold change) | ≥1. The heatmap of the DEGs was shown in Figure 1A. Meanwhile, a total of 1,811 IRGs were extracted from the ImmPort database. Then, 476 differentially expressed IRGs were identified using the intersect function of Venn diagram (Figure 1B). The heatmap of the differentially expressed IRGs was shown in Figure 1C. We then performed GO and KEGG enrichment analyses to further explore the biological function of these IRGs. As is shown in Figure 1D, GO enrichment analysis found that these IRGs are mainly enriched in immune-related crosstalk, including complement activation, humoral immune response, and leukocyte migration. KEGG pathway enrichment analysis showed that these genes are mainly enriched in several areas of tumor-related pathway, including the Rap 1 signaling pathway, MAPK signaling pathway, and NF-κB signaling pathway (Figure 1E).

### Construction of Risk Assessment Signature

**FIGURE 2:**
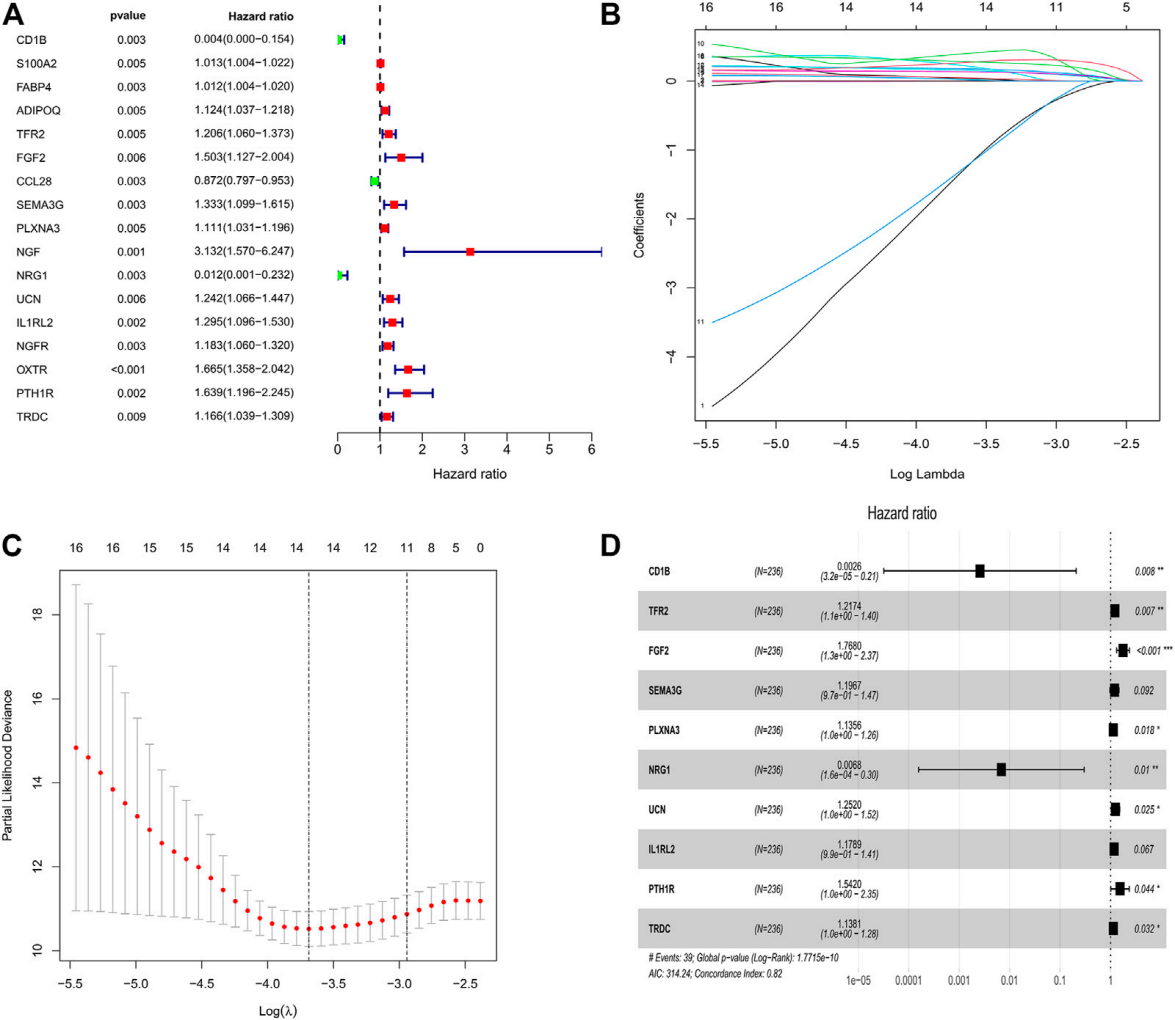
Identification of survival related IRGs in patients with CRC. **(A)** Univariate Cox regression models identified 17 IRGs associated with prognosis of CRC patients. **(B, C)** Lasso regression models identified 14 IRGs. **(D)** Ten IRGs were identified to construct a novel prognostic signature based on multivariate Cox regression models.

First, the entire TCGA cohort (*n* = 472) was randomly divided into the training cohort (*n* = 236) and internal validation cohort (*n* = 236), and the clinical parameters of the CRC patients in each cohort are shown in Table 1. Then, we used the expression profiles of the differentially expressed IRGs in the training cohort to construct a survival-related signature. In total, 17 IRGs were identified to be associated with the prognosis of patients with CRC using univariate Cox regression analysis (*p* < 0.01, Figure 2A) and 14 of which were filtered out using a LASSO regression analysis with a 10-fold cross validation (Figures 2B,C). Finally, 10 IRGs were identified as independent prognostic factors and were selected for constructing the IRG signature (Figure 2D; Table 2). The risk score for each sample was calculated based on the expression level and Cox regression coefficient of these 10 genes. Risk score = (−5.96137 × EXP_CD1B_) + (0.19671 × EXP_TFR2_) + (0.56983× EXP_FGF2_) + (0.17961 × EXP_SEMA3G_) + (0.12713 × EXP_PLXNA3_) + (−4.98459 × EXP_NRG1_) + (0.22476 × EXP_UCN_) + (0.16455 × EXP_IL1RL2_) + (0.43311 × EXP_PTH1R_) + (0.12939 × EXP_TRDC_).

**TABLE 1:** Clinical features of the patients with CRC in each cohort.

Variables	Training cohort (*n* = 236)	Testing cohort (*n* = 236)	*P*	Entire TCGA cohort (*n* = 472)
Age (years)				
≤65	98	111	0.2661	209
>65	138	125		263
Gender				
Female	108	103	0.7111	211
Male	128	133		261
Survival status				
Alive	197	193	0.7155	390
Dead	39	43		82
Tumor invasion (T)				
T1	7	6	0.8146	13
T2	43	38		81
T3	161	163		324
T4	25	28		53
Unknown	0	1		1
Lymph node (N)				
N0	135	137	0.7794	272
N1	61	58		119
N2	40	40		80
Unknown	0	1		1
Metastasis (M)				
M0	202	198	0.6814	400
M1	32	34		66
Unknown	2	4		6
Tumor stage				
Stage I	40	40	0.9899	80
Stage II	91	86		177
Stage III	66	68		134
Stage IV	32	34		66
Unknown	7	8		15

**TABLE 2:** Multivariate Cox results of immune-related mRNAs based on TCGA data.

Gene	Coefficient (β)	HR	95% CI of HR
CD1B	−5.96137	0.0026	3.20e-05-0.2075
TFR2	0.19671	1.2174	1.0552–1.4046
FGF2	0.56983	1.7680	1.3191–2.3695
SEMA3G	0.17961	1.1968	0.9712–1.4747
PLXNA3	0.12713	1.1356	1.0223–1.2614
NRG1	−4.98459	0.0068	0.0002–0.2990
UCN	0.22476	1.2520	1.0281–1.5248
IL1RL2	0.16455	1.1789	0.9885–1.4059
PTH1R	0.43311	1.5420	1.0108–2.3526
TRDC	0.12939	1.1381	1.0109–1.2813

HR, hazard ratio; CI, confidence interval.

### Prognostic Evaluation by Risk Assessment Signature

**FIGURE 3:**
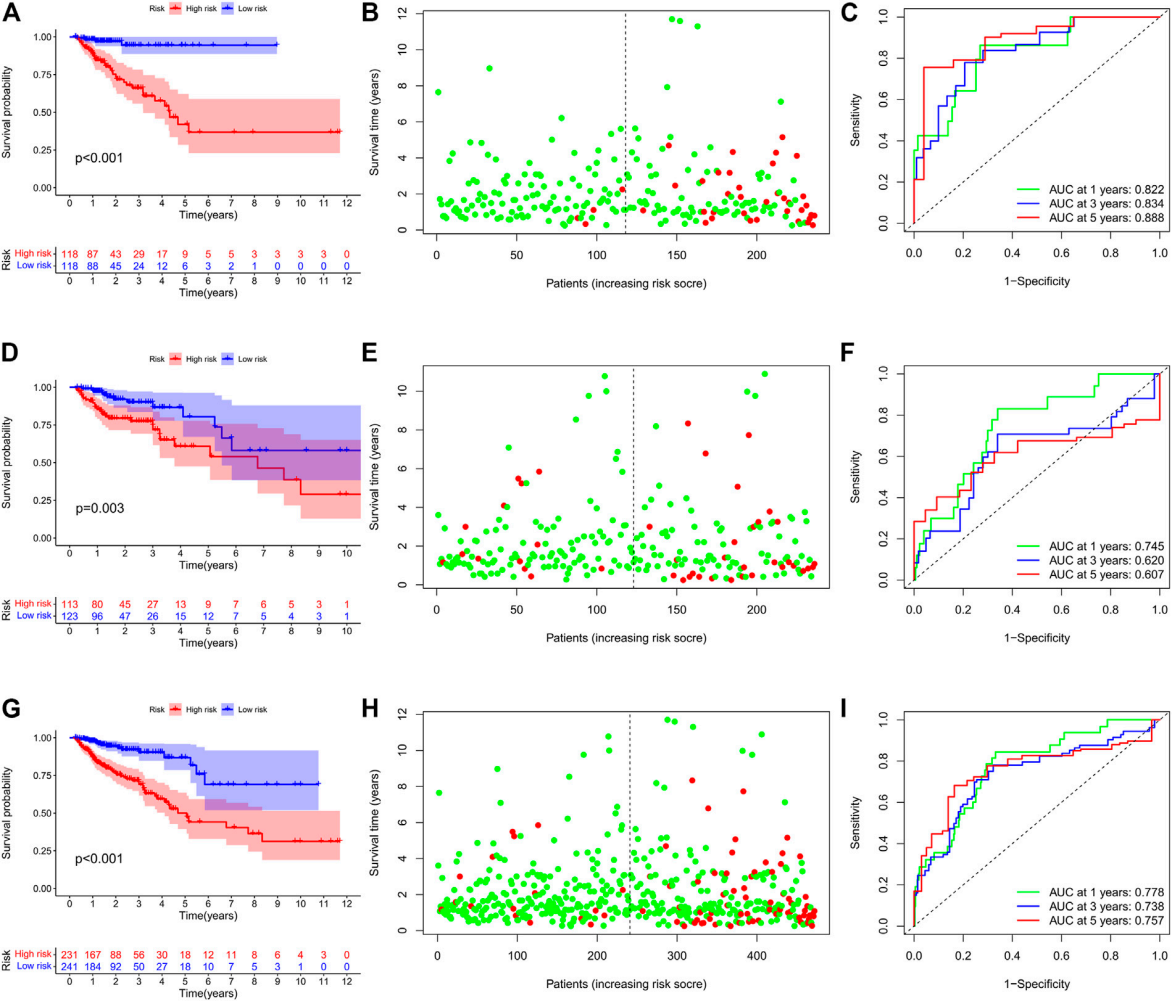
Prognostic value of the IRG signature in human CRC. **(A)** Kaplan–Meier survival curve, **(B)** risk plot, and **(C)** ROC curve of the IRG signature in the training cohort. **(D)** Kaplan–Meier survival curve, **(E)** risk plot, and **(F)** ROC curve of the IRG signature in the internal validation cohort. **(G)** Kaplan–Meier survival curve, **(H)** risk plot, and **(I)** ROC curve of the IRG signature in the entire TCGA cohort.

Based on the cutoff value of 1.763 for the risk score, patients were divided into the low- (*n* = 118) and high-risk (*n* = 118) groups. The Kaplan–Meier analysis with the log-rank test found that patients in the high-risk group presented worse prognosis than those in the low-risk group (Figure 3A). CRC patients appeared to have an increased mortality rate with the increase of risk scores according to the risk plot (Figure 3B). The time-ROC curve showed that the AUC of 1-, 3-, and 5-year survival were 0.822, 0.834, and 0.888, respectively (Figure 3C). Then, we further validated the immune signature in the internal validation and the entire TCGA cohorts. The prognosis of CRC patients in the high-risk group was significantly worse than that in the low-risk group (Figure 3D). The patients with CRC have an increased mortality rate with the increase of risk scores according to the risk plot (Figure 3E). The AUC values of 1-, 3-, and 5-year survival in the internal validation cohort were 0.745, 0.620, and 0.607, respectively (Figure 3F). Similarly, in the entire TCGA cohort, patients in the high-risk group presented worse prognosis than those in the low-risk group (Figure 3G). The mortality rate of patients was increased with the increase of risk scores according to the risk plot (Figure 3H), and the AUC for 1-, 3-, and 5-year survival was 0.778, 0.737, and 0.757, respectively (Figure 3I). These aforementioned results indicated that the IRG signature was competent for predicting the prognosis of human CRC.

**FIGURE 4:**
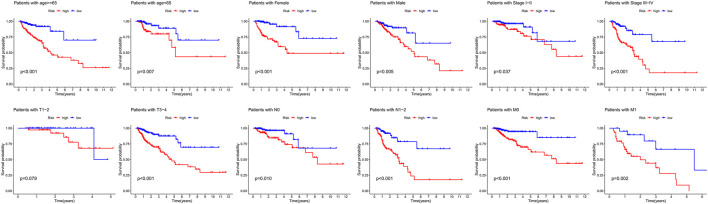
Survival differences between high- and low-risk CRC patients stratified by clinical factors.

We performed stratification analysis to further determine the widespread utility of the IRG signature based on the clinicopathological variables. As shown in Figure 4, we found that the IRG signature has predictive significance for different subgroups, and the prognosis of patients in the low-risk group was significantly superior to that of patients in the high-risk group. Taken all above, these findings indicate that the IRG signature exerts vital roles in predicting the prognosis of CRC patients.

**FIGURE 5:**
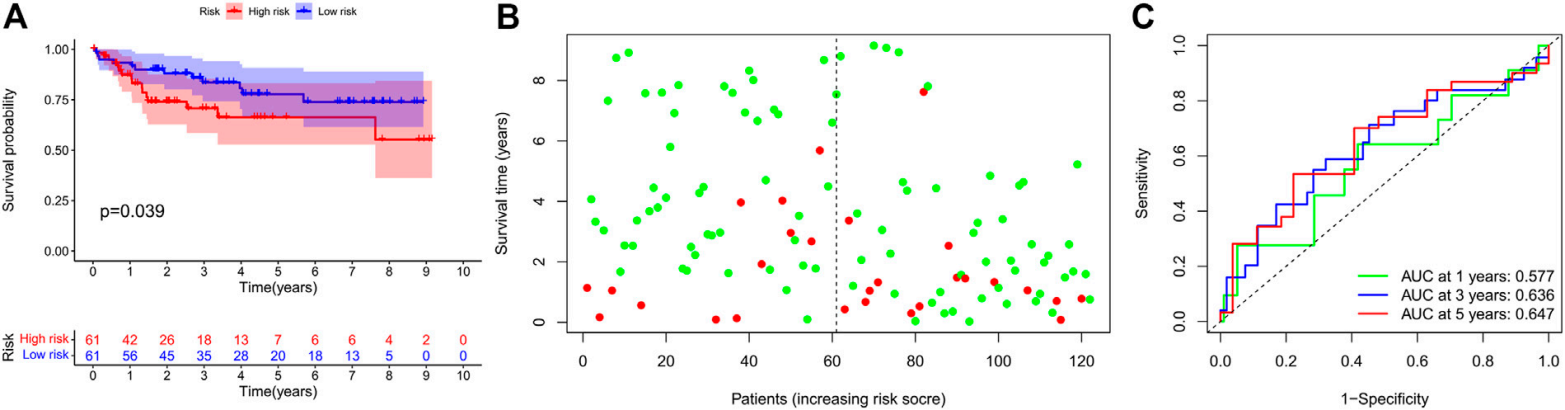
Prognostic value of the IRG signature in external validation cohort. **(A)** Kaplan–Meier survival curve, **(B)** risk plot, and **(C)** ROC curve of the IRG signature in the GEO cohort.

Finally, we further validated the prognostic value of the TRG signature in an independent cohort from the GEO project. Similarly, survival analysis in 122 CRC patients also found that the high-risk score indicated poor survival outcomes (Figures 5A,B). Correspondingly, the 1-, 3-, and 5-year AUC in predicting prognosis was 0.577, 0.636, and 0.647, respectively (Figure 5C).

### Correlation Between the Signature and the Clinicopathologic Parameters in CRC

**FIGURE 6:**
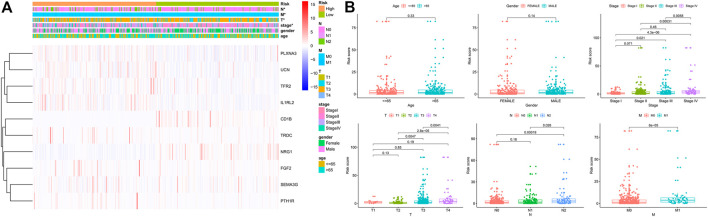
Correlation between the risk score and different clinicopathological parameters. **(A)** The heatmap shows the distribution of clinicopathological parameters and the expression of the IRGs between the low- and high-risk groups. Chi-square test was used for correlation between clinical and risk. *p < 0.05. **(B)** Scatter diagram showed that high-risk score levels correlated with advanced pathological stages.

We performed a series of Shi-square tests and Wilcoxon tests to determine the correlations between the risk score and the clinicopathological parameters in patients with CRC. As shown in the strip chart (Figure 6A) and scatter diagrams (Figure 6B), The risk scores were found to be significantly associated with patients’ clinicopathologic parameters, including T classification, N classification, M classification, and AJCC tumor stage. These aforementioned results indicated that the high-risk score was significantly correlated with more aggressive clinicopathological characteristics.

### Construction of the Prognostic Nomogram

**FIGURE 7:**
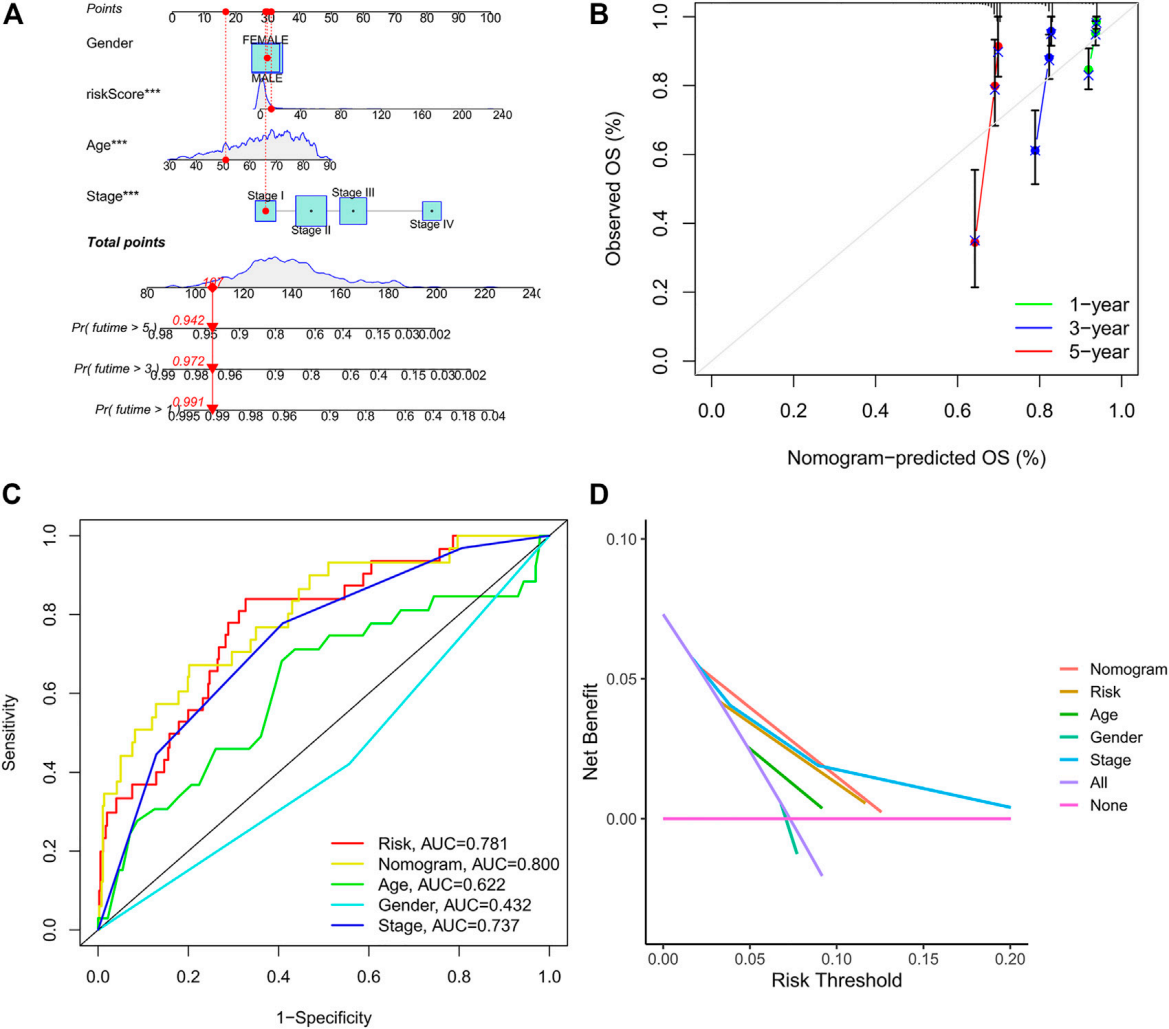
Construction of the prognostic nomogram. **(A)** The nomogram of 1-, 3-, and 5-year overall survival based on risk score, age, gender, and TNM stage. **(B)** Calibration plot of nomogram for predicting probabilities of 1-, 3-, and 5-year survival of patients with CRC. **(C)** A comparison of ROC curves showed the superiority of the nomogram and IRG signature. **(D)** Decision curve analysis of nomogram, IRG signature, and other clinicopathological features.

The age, gender, stage, and risk score were integrated to construct a nomogram to predict the survival probability of CRC patients at 1, 3, and 5 years (Figure 7A). The calibration plot showed that the nomogram performed well compared against the performance of an ideal model (Figure 7B). The ROC curve showed that the AUC values corresponding to nomogram, risk score, stage, gender, and age were 0.800, 0.781, 0.737, 0.436, and 0.622, respectively (Figure 7C). In addition, the DCA curve proved that the nomogram provided better net benefits than other prognostic factors (Figure 7D). These results indicated that the nomogram signified satisfied accuracy in predicting the prognosis of CRC patients.

### Comparison of the IRG Signature With Other Prognostic Models

**FIGURE 8:**
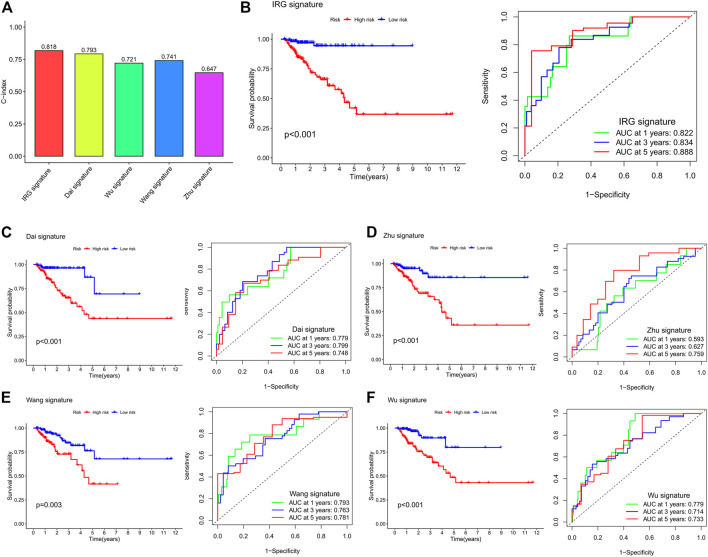
Comparison of the IRG signature with previously identified prognostic models. **(A)** The C-index of the IRG signature and previously identified prognostic models. **(B)** Kaplan–Meier survival curve and ROC curve of the IRG signature. **(C)** Kaplan–Meier survival curve and ROC curve of Dai et al.’s signature. **(D)** Kaplan–Meier survival curve and ROC curve of Zhu et al.’s signature. **(E)** Kaplan–Meier survival curve and ROC curve of Wang et al.’s signature. **(F)** Kaplan–Meier survival curve and ROC curve of Wu et al.’s signature. The survival curve is used to analyze the difference in prognosis between the low- and high-risk groups, and the AUC value of the ROC curve is used to evaluate the predictive ability.

We compared our IRG signature with previously identified models to determine whether the predictive efficiency of our signature is higher than others. As shown in Figure 8A, the C-index of our signature is 0.818, which is higher than that in other models. The Kaplan–Meier survival curve showed that the prognosis of the low- and high-risk groups significantly differed across these signatures (Figures 8B–F). The ROC curve showed that the AUC values of the 1-, 3-, and 5-year survival for our signature are 0.822, 0.834, and 0.888, respectively (Figure 8B). The AUC values of the 1-, 3-, and 5-year survival for Dai et al. signature are 0.779, 0.799, and 0.748, respectively (Figure 8C). The AUC values of the 1-, 3-, and 5-year survival for Zhu et al. signature are 0.593, 0.627, and 0.759, respectively (Figure 8D). Wang et al. signature showed that the AUC values of the 1-, 3-, and 5-year survival are 0.793, 0.763, and 0.781, respectively (Figure 8E). Wu et al. signature showed that the AUC values of the 1-, 3-, and 5-year survival are 0.779, 0.714, and 0.733, respectively (Figure 8F). By comparing with previously constructed signatures, we found that the predictive efficiency of our IRG signature is higher than that of other models.

### Estimation of Tumor-Infiltrating Immune Cells and Immunotherapeutic Biomarkers With Risk Assessment Signature

**FIGURE 9:**
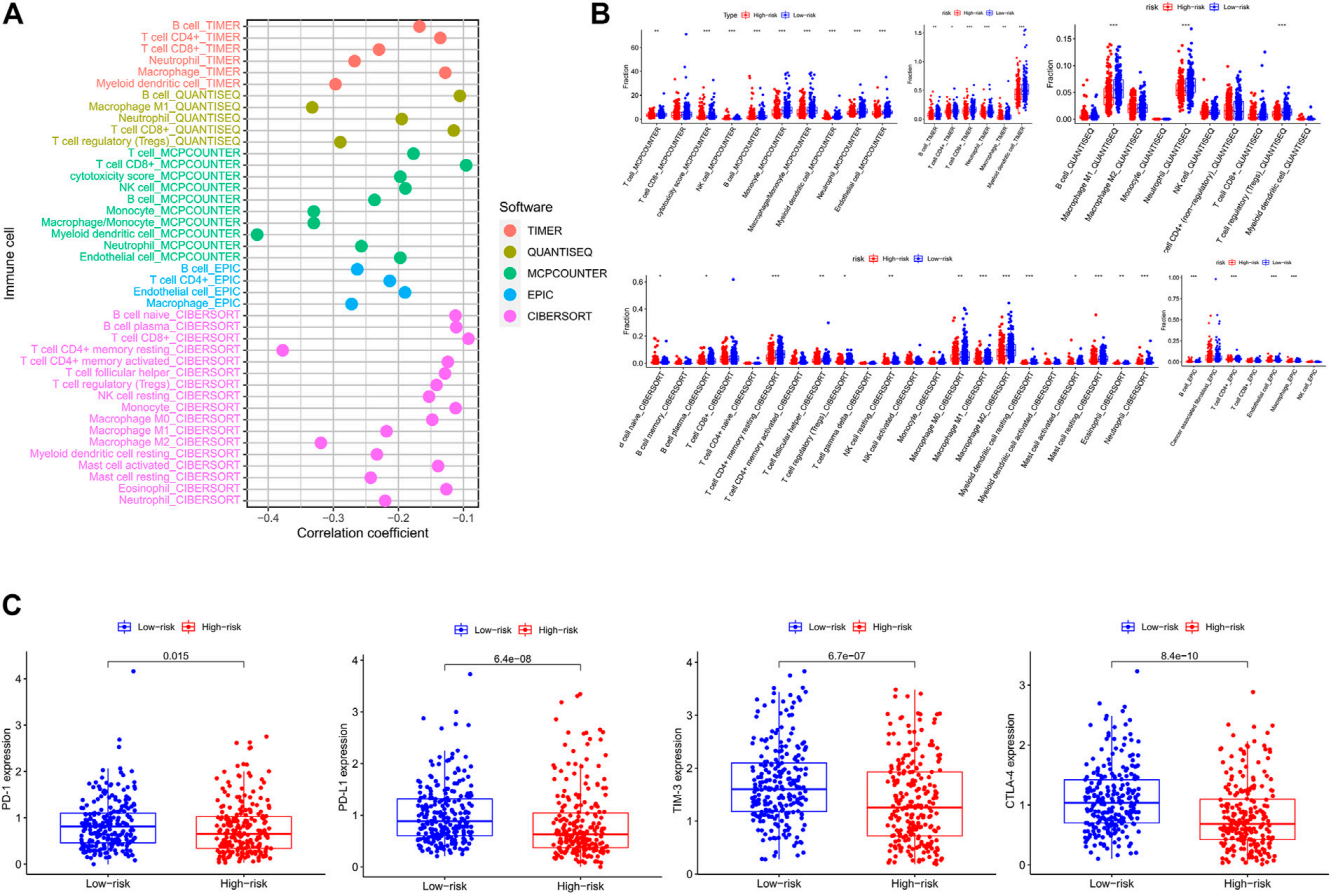
Estimation of tumor-infiltrating immune cells and immune checkpoint biomarkers by the risk assessment signature. **(A)** Lollipop plot showing the correlations between the abundance of different TIIC and risk score calculated by Spearman correlation analysis. **(B)** The difference in abundances of different TIIC between the low- and high-risk groups based on distinct algorithms. **(C)** The difference in expression levels of immune checkpoint biomarkers between the low- and high-risk groups.

In order to explore whether the IRG signature was associated with the immune characteristics of CRC, we applied five algorithms to estimate the abundance of TIICs. As shown in the lollipop diagram (Figure 9A), Spearman correlation analysis revealed that the risk score was significantly negatively correlated with the infiltration levels of multiple TIICs, including CD4^+^ T cells, CD8^+^ T cells, NK cells, macrophages, B cells, and neutrophils. The box plot showed that the abundance of these TIICs was significantly higher in the low-risk group than that in the high-risk group (Figure 9B). In addition, we further explored the expression of immunotherapeutic biomarkers in low- and high-risk groups. As shown in Figure 9C, compared to the high-risk group, CRC patients in the low-risk group express higher levels of immunotherapeutic biomarkers, including PD-1, PD-L1, TIM-3, and CTLA-4. These results indicated that CRC patients in the low-risk group had an immune “hot” phenotype and were more likely to benefit from immunotherapy. That is, to say, the IRG signature can predict the immune characteristics of human CRC.

## Discussion

Cancer evolution is a complicated progression which involves diverse processes from the accumulation of genetic alterations to epigenetic modulations and chromosomal abnormalities (Baviskar et al., 2021). It is widely accepted that genetic alterations such as mutations in oncogenes and tumor suppressor genes (e.g., KRAS, NRAS, BRAF, PIK3CA, and TP53) directly contribute to tumorigenesis (Testa et al., 2018). In addition, studies have also indicated that epigenetic modifications are involved in the onset and progression of multiple human diseases (Bauer et al., 2016). Epigenetics focused on heritable alterations in gene expression that do not arise permanent alterations in the primary DNA sequence, including DNA methylation, genomic imprinting, gene silencing, and RNA editing. Accumulating evidence has reported that epigenetic alteration has a central role in the pathogenesis of various types of cancers (Gu et al., 2015; Jung et al., 2020). Recently, the rapidly developed next-generation sequencing (NGS) and whole-genome bisulfite sequencing (WGBS) provide us more convenient platforms to explore the genetic and epigenetic alterations in many types of human diseases, including CRC (Wang et al., 2013; Ratovomanana et al., 2021). Nevertheless, the pathogenesis of CRC remains largely unclear and deserves further clarification.

Tumorigenesis is affected not only by its intrinsic characteristics but also by extrinsic TME, and growing evidence has indicated the significant role of the TME in predicting tumor progression and prognosis (Wang et al., 2019). Immune cells are important components of TME, and dysfunction of the immune system in cancer patients will lead to immune escape of cancer cells, thereby promoting the initiation and progression of cancer (Yang et al., 2019). Recent studies have indicated that immunotherapy is an effective strategy for the treatment of multiple solid tumors. However, available evidence suggests that most CRC patients are insensitive to existing immunotherapies due to tumor heterogeneity and lack of effective biomarkers. Therefore, identifying novel biomarkers to predict the immune characteristics and therapeutic sensitivity of human CRC is very important.

First, we obtained the transcriptome sequencing data of CRC from TCGA, and a total of 476 differentially expressed IRGs were identified. Then, we utilized GO and KEGG enrichment analyses to explore the potential function of these IRGs. GO analysis found that these IRGs were mainly enriched in immune-related biological processes, such as complement activation, humoral immune response, and leukocyte migration. In addition, KEGG pathway enrichment analysis revealed that these genes were mainly enriched in the tumor-related pathway, including the Rap 1 signaling pathway, MAPK signaling pathway, and NF-κB signaling pathway. Among the enriched pathways, dysregulation of Rap 1 signaling pathway has been reported to promote the cell proliferation and apoptosis of pancreatic cancer (Yao et al., 2019). The MAPK signaling pathway has also been reported to regulate many cellular functions, including cell proliferation and apoptosis, and dysregulation of MAPK signaling pathway participating in the initiation and progression of CRC (Slattery et al., 2018). There is also increasing evidence that the NF-κB signaling pathway was abnormally activated in various cancer cell lines including CRC, with the silencing of NF-κB signaling pathway significantly inhibiting cell proliferation and migration of CRC *in vitro* (Soleimani et al., 2020). Therefore, these differentially expressed IRGs play important roles in human CRC.

Subsequently, an immune-related signature comprising10 IRGs was constructed based on univariate Cox regression, lasso regression, and multivariate Cox regression analyses in the TCGA project. Some of the IRGs involved in the present signature have been reported previously to play an important role in the carcinogenesis of multiple cancer types, including CRC. For example, CD1B belongs to the group 1 CD1 family of glycoproteins, which has been shown to present lipid antigens on the surface of antigen presenting cells (APCs) in humans (Shahine, 2018). A previous study has reported that the expression patterns of CD1 molecules in cancer cells differ from those in normal cells, and dysregulation of CD1B was associated with the prognosis of patients with prostate cancer (Lu et al., 2020). TFR2 encodes a single-pass type II membrane protein, which plays a key role in the regulation of iron homeostasis. Studies have reported that TFR2 is highly expressed in CRC cells, and found that this was associated with ERK1/ERK2 phosphorylation (Calzolari et al., 2009). FGF2 is a member of fibroblast growth factor family, which has been reported to participate in several tumor-related pathways, such as cell growth, differentiation, and angiogenesis (Akl et al., 2016). Studies have shown that FGF2 is significantly overexpressed in CRC tissues compared to normal tissues, and CRC patients with high FGF2 expression showed poorer prognosis than those with low expression (Caiado et al., 2020). NRG1 gene is an emerging, potentially actionable oncogenic driver that can promotes pathologic signaling such as MAPK and ERBB pathways (Jonna et al., 2019). In addition, NRG1 was previously found to play a significant role in primary cetuximab resistance of CRC (Bray et al., 2019). UCN is mainly expressed in reproductive organs and urinary systems, and dysregulation of UCN can affect the invasion and metastasis of endometrial cancer (Wen et al., 2020). PTH1R, also known as PTHR1, is a crucial regulator of calcium homeostasis in humans. The overexpression of PTH1R has been identified as a potential biomarker for liver metastasis of CRC (Liu et al., 2019). These previous studies illustrated that IRGs can serve as potential biomarkers for anti-cancer therapy and have a significant influence on the prognosis of cancer patients. However, biomarkers that can effectively predict the prognosis of CRC patients are still lacking. The present study involved these prognostic IRGs and constructed a signature to predict the prognosis of CRC patients. Survival analysis revealed that the IRG signature could accurately predict the overall survival of the patients with CRC in the training cohort, the internal validation cohort, and the entire TCGA cohort. Importantly, the external validation cohort further confirmed that the IRG signature serves as a reliable predictor for the prognosis of CRC patients.

In the last few years, anti-cancer immunotherapy has gained unprecedented success as an alternative cancer therapeutic strategy for many types of solid tumors through reactivating the host immune system. Indeed, TIICs have been recognized as a strong prognostic biomarker in human CRC despite the immune landscape variation across different consensus molecular subtypes (CMSs) or mismatch repair (MMR) phenotypes (Picard et al., 2020; Bruni et al., 2020). For example, previous studies have reported that CD4^+^ T cells can secrete IL-1, IL-6, IFN-γ, and other cytokines in TME, thereby activating other immune cells to eliminate the intracellular pathogens and tumor cells, but the immunity of CD4^+^ T cells is usually suppressed by CRC cells via activating the Wnt signaling pathway (Lin et al., 2020; Sun et al., 2017). NK cells exert cytotoxic effects by secreting TNF, perforin, and granzyme, and tumor-associated NK cells have been found to associate with increased survival time in patients with CRC (Bruni et al., 2020; Lin et al., 2020; Coca et al., 1997). Macrophages M2, the majority phenotypes of macrophages present in the TME, are induced by IL-4 and IL-13 and showed tumor-promoting activity in various solid tumors, including CRC (Popēna et al., 2018). Moreover, previous studies have illuminated that macrophages M2 could regulate 5-fluorouracil resistance of CRC cells through the epithelial–mesenchymal transition (EMT) program, PI3K/AKT pathway, and caspase-mediated apoptosis (Wei et al., 2019). The activated CD8^+^ T cells can induce specific destruction of cancer cells by releasing lytic components, it is the major killer of cancer cells in the anti-cancer immune response and constitutes the backbone of cancer immunotherapy (Raskov et al., 2021; Titu et al., 2002). A high degree of CD8^+^ T cells infiltrating abundance has been reported to be significantly correlated with more favorable prognosis in patients with CRC (Tada et al., 2016). In addition, dendritic cells, mast cells, and neutrophils have also been reported to associate with the initial and progression of CRC (Bruni et al., 2020). In the present study, we found that the IRG signature was significantly correlated with the abundance of multiple TIICs, including CD4^+^ T cells, CD8^+^ T cells, NK cells, macrophages, B cells, and neutrophils. CRC patients in the low-risk group infiltrated with a higher density of TIICs than those in the high-risk group, which means that the natural anti-cancer immune response in the low-risk group was more active than that in the high-risk group. Immune checkpoint molecules are inhibitory receptors which are expressed on the surface of immune cells to regulate the immune response. Studies have reported that the expression of immune checkpoint biomarkers can be used as positive predictive biomarkers for the efficacy of immunotherapy (Bethmann et al., 2017). Thus, we further explored the association between the IRG signature and the expression of immune checkpoint biomarkers and found that CRC patients in the low-risk group expressed a significantly higher level of immune checkpoint biomarkers than those in the high-risk group. To sum up, these aforementioned findings suggested that the immune landscape of low-risk tumors was defined as “immune active” with high infiltration of TIICs, while the immune landscape of high-risk tumors can be defined as “immune desert” with a poor intratumoral immune response characterized by the low densities of TIICs. That is, to say, the present IRG signature can predict the immune characteristics of CRC, and patients in the low-risk group are more likely to benefit from anti-tumor immunotherapy than those in the high-risk group.

Inevitably, there are several limitations in the present study. First, all the data analyzed in the present study were obtained from the online database, and the amount of data included is not large enough, so the analysis results may have some deviation. Second, during the construction of IRG signature, we took the prognostic value as the only filter criterion, which may inevitably omit some important immune genes. Thus, we will collect clinical samples and expand the sample size to further validate the predictive ability of the IRG signature in the future, the evaluation of which would be a time-consuming process.

## Conclusion

In conclusion, the present study constructed a prognostic signature which is composed of 10 IRGs that could accurately predict the prognosis of patients with CRC and might help to distinguish those who could benefit from anti-tumor immunotherapy.

## Data Availability

The original contributions presented in the study are included in the article/Supplementary Material, further inquiries can be directed to the corresponding author.
